# Anchored Drill-Assisted Localization for Percutaneous Endoscopic Cervical Keyhole Surgery in the Treatment of Cervical Spondylotic Radiculopathy: A Technical Report and Preliminary Efficacy Evaluation

**DOI:** 10.3390/jcm15197423

**Published:** 2026-09-24

**Authors:** Zhuangzhuang Tan, Bin Xie, Jiajun Cheng, Yutao Wang, Hui Ren, Weibo Yu, Xiaobing Jiang

**Affiliations:** 1 The Second Affiliated Hospital of Guangzhou Medical University, Guangzhou 510261, China; 2025210906@stu.gzhmu.edu.cn (Z.T.); 2024311349@stu.gzhmu.edu.cn (B.X.); 15179312807@163.com (J.C.); 2020111311@stu.gzhmu.edu.cn (Y.W.); renhuispine@163.com (H.R.); 2 The Second Clinical School, Guangzhou Medical University, Guangzhou 510180, China

**Keywords:** cervical spondylotic radiculopathy, keyhole, posterior cervical foraminotomy, anchored drill, surgical navigation, vertebral artery safety

## Abstract

**Objectives:** This study presents a novel anchored drill-assisted localization technique for percutaneous endoscopic cervical keyhole surgery in single-level cervical spondylotic radiculopathy (CSR). This technical report describes its feasibility, preliminary efficacy, and safety. **Methods:** We retrospectively analyzed 40 consecutive patients with single-level CSR treated with this technique (July 2023–August 2025). Operative time, blood loss, hospital stay, and complications were recorded. Decompression safety was evaluated on postoperative computed tomography (CT) by measuring the distance between the bone resection margin and the intervertebral foramen. Clinical outcomes were assessed using the visual analog scale (VAS) for neck and arm pain, the Japanese Orthopaedic Association (JOA) score, the Neck Disability Index (NDI), and modified MacNab criteria. **Results:** No major complications occurred. Minor complications included transient axial neck pain in 3 patients (7.5%) and persistent axial neck pain in 1 patient (2.5%). After a median follow-up of 15.2 (12.2–26.1) months, all clinical scores improved significantly (all *p* < 0.001). The mean JOA score increased from 13.8 ± 2.0 to 16.5 ± 1.1, and mean NDI decreased from 45.6 ± 8.9% to 9.8 ± 8.2%. Neck and arm VAS decreased from 5.4 ± 1.8 and 5.4 ± 2.1 to 0.7 ± 1.2 and 0.8 ± 1.0, respectively. The excellent/good rate by modified MacNab criteria was 97.5% (39/40). **Conclusions:** The anchored drill-assisted technique may enhance spatial orientation, potentially reduce fluoroscopy use, and establishes a clear safety boundary. This preliminary report demonstrates favorable safety and efficacy in treating single-level CSR. Prospective comparative studies are warranted.

## 1. Introduction

Cervical Spondylotic Radiculopathy (CSR) is a prevalent clinical syndrome caused by compression or irritation of cervical nerve roots, typically due to degenerative disc disease, uncovertebral joint osteophytosis, or hypertrophy of the posterior longitudinal ligament [1,2,3]. Its hallmark symptoms—neck pain with radiating arm pain, numbness, and paresthesia along the affected dermatome—significantly impair patients’ quality of life and functional capacity. With an aging population and changing lifestyles, the prevalence of CSR continues to rise. For patients with refractory symptoms unresponsive to conservative management, surgical intervention remains the mainstay for effective neural decompression.

Anterior Cervical Discectomy and Fusion (ACDF), renowned for its technical maturity and reliable outcomes, is widely considered the “gold standard” surgical treatment for CSR [3]. However, its benefits come at the cost of segmental motion, which may lead to pseudarthrosis, implant-related complications, and accelerated adjacent segment degeneration (ASD), particularly when multiple levels are fused [3,4,5,6,7]. To preserve motion, Posterior Cervical Foraminotomy (PCF) has been developed. With advancements in minimally invasive spine instrumentation, open PCF has evolved into microendoscopic or tubular-assisted keyhole surgery. This technique achieves direct nerve root decompression through a minimal bony window while maximizing preservation of cervical segmental mobility, making it an ideal choice for unilateral foraminal stenosis or lateral disc herniation [8,9,10].

Nevertheless, endoscopic keyhole surgery presents significant challenges, including a steep learning curve, a constrained operative field, difficulties with intraoperative localization, risk of excessive facet joint resection, and the potential for vertebral artery injury [11,12,13,14,15].

To address these limitations, this study introduces a percutaneous endoscopic cervical keyhole technique augmented by anchored drill-assisted localization. The core innovation involves implanting a specially designed anchored drill at the superolateral edge of the pedicle of the caudal vertebra in the target segment. This creates an immovable, deep intraosseous reference point. This artificial landmark may fundamentally eliminate wrong-level surgery and, through a “set-once, guide-throughout” paradigm, may drastically reduce the need for intraoperative fluoroscopy, thereby lowering radiation exposure. Furthermore, by capitalizing on the constant anatomical relationship between this anchor point and the vertebral artery, the technique predefines an operative trajectory that is obliquely directed superomedially from the anchor point. This establishes a clear “safety boundary” for decompression, proactively mitigating the risk of vertebral artery injury, minimizing iatrogenic bony destruction, and ultimately preserving the long-term stability of the cervical motion segment. We report the feasibility, preliminary clinical outcomes, and safety profile of this technique.

## 2. Materials and Methods

### 2.1. Study Design

We retrospectively reviewed the clinical data of 40 patients with single-level CSR who underwent anchored drill-assisted percutaneous endoscopic cervical keyhole surgery between July 2023 and August 2025. All procedures were performed by a single senior spine surgeon with over 20 years of experience in minimally invasive spine surgery using a monaxial spinal endoscopy system (Shandong Weigao Group Medical Polymer Co., Ltd., Weihai, Shandong, China). Given the descriptive nature of this technical report, no control group was included. All outcome measures are reported as preliminary findings. Sample size was not determined a priori based on power calculation, given the exploratory and descriptive nature of this technical report. The 40 patients represent consecutive cases who met the inclusion criteria and underwent the anchored drill-assisted procedure during the study period (July 2023 to August 2025). Such a sample size is acceptable for an exploratory preliminary technical report.

### 2.2. Indications and Contraindications

Inclusion criteria were: (1) MRI and CT evidence of paracentral or foraminal disc herniation with or without osteophytes at any level from C2-C3 to C7-T1, correlating with the symptomatic nerve root; (2) Unilateral radicular symptoms (radiating pain with or without sensory changes in the corresponding dermatome); (3) Symptomatic unilateral foraminal stenosis; (4) Failure of at least 3 months of structured conservative therapy or progressive symptom deterioration; (5) Candidate for endoscopic cervical keyhole surgery.

Exclusion criteria included: (1) Cervical segmental instability, kyphosis, or scoliosis requiring fusion; (2) Severe central canal stenosis with myelopathic symptoms; (3) Epidural tumors or other space-occupying lesions causing radiculopathy; (4) Severe comorbidities precluding surgery; (5) Inability to complete postoperative follow-up.

### 2.3. Surgical Technique

The anchored drill used in this study is a custom-designed prototype device, manufactured by Shandong Weigao Group Medical Polymer Co., Ltd. (Weihai, Shandong, China). It has not been commercialized and is not available as a mass-produced product with a trade name or model number. The drill features a non-threaded tip with a depth-limiting safety structure; the tip is 4 mm in height and 2 mm in diameter. The device is made of medical-grade stainless steel. Detailed specifications and design drawings are available from the corresponding author upon reasonable request. The anchored drill-assisted procedure was performed in four sequential steps: (1) patient positioning, (2) precise implantation of the anchored drill (technical core), (3) endoscopic decompression, and (4) verification of decompression and wound closure. A custom-designed anchored drill was used for localization (Figure 1). The following description details the procedure for a representative case with a target level of C5–C6.

#### 2.3.1. Anesthesia and Positioning

General anesthesia was induced, and the patient was positioned prone. The head was rigidly fixed using a three-pin Mayfield head holder (Shandong Weigao Group Medical Polymer Co., Ltd., Weihai, Shandong, China). The operating table was adjusted to align the target intervertebral space perpendicular to the floor, facilitating adherence to the “double perpendicular” principle during instrument insertion: on lateral fluoroscopy, the long axis of the anchored drill was maintained perpendicular to both the plane of the lamina and the horizontal ground (Figure 2).

#### 2.3.2. Approach and Anchored Drill Implantation

Under biplanar C-arm fluoroscopy (Jiangsu First-Imaging Medical Equipment Co., Ltd., Nantong, Jiangsu, China), the C5–C6 level was identified. The skin entry point, typically approximately 2 cm lateral to the C6 spinous process, was marked. A puncture needle was advanced under fluoroscopic guidance toward the superolateral edge of the C6 pedicle’s surface projection, targeting the junction of the C6 lamina and lateral mass. A guidewire was inserted, followed by a 7–10 mm skin incision. Sequential dilation was performed using a “pencil-tip” dilator (Shandong Weigao Group Medical Polymer Co., Ltd., Weihai, Shandong, China), after which the working cannula was placed. Through this cannula, the depth-limited anchored drill was implanted at the superolateral edge of the C6 pedicle and gently tapped into the bone (Figure 3). During the anchored drill implantation and localization process, the median number of fluoroscopic shots was 5 (range, 4–8). Final fluoroscopic images confirmed the accurate position of the drill. A stable endoscopic working channel was established, after which the anchored drill was removed. The anchored drill was implanted through the working cannula and gently tapped into the superolateral edge of the pedicle of the caudal vertebra until the depth-limiting structure reached the cortical surface. After fluoroscopic confirmation of accurate placement, the endoscopic working channel was established, and the drill was immediately removed. The drill remained in situ only briefly (typically 1–2 min) and was not left in place throughout the entire procedure.

#### 2.3.3. Endoscopic Decompression

Under continuous endoscopic visualization, soft tissue within the channel was cleared to expose the anchor point. This point served as the fixed inferolateral safety boundary. Exploration superomedially from this point reliably identified the “V” point of the C5–C6 interlaminar space (Figure 4A,B). Using a high-speed burr (Shandong Weigao Group Medical Polymer Co., Ltd., Weihai, Shandong, China), a C5 laminotomy and partial inferior facetectomy were performed starting at the “V” point until the cranial insertion of the ligamentum flavum was visualized (Figure 4C). Subsequently, the C6 lamina and partial superior facet were drilled to expose the pedicle (Figure 4D). After sufficient bony removal, the underlying ligamentum flavum was resected, revealing the lateral dural sac and the exiting nerve root dorsally. The nerve root was then traced laterally and inferiorly to identify the medial border of the pedicle. Decompression of the nerve root axilla was performed along this border (Figure 4E). Consistent with the predefined safety boundary, all instrumentation was confined to the medial and cephalad aspects of the anchor point.

#### 2.3.4. Closure

After confirming adequate nerve root decompression, pulsation, and hemostasis (Figure 4F), the endoscope and working cannula were removed. The incision was closed in layers. No postoperative drains were placed, as meticulous hemostasis was achieved endoscopically and no significant epidural bleeding was observed.

### 2.4. Clinical and Radiological Assessment

Clinical and demographic data were collected retrospectively. Clinical outcomes were assessed preoperatively and at the final follow-up using the VAS for neck and arm pain, the JOA score, the NDI, and the modified MacNab criteria. Complications were defined as any adverse event occurring within 30 days postoperatively, including major complications (e.g., dural tear, nerve root injury, vertebral artery injury, epidural hematoma requiring evacuation, surgical site infection) and minor complications (e.g., transient worsening of radicular symptoms, transient axial neck pain, superficial wound issues).

On postoperative CT (obtained 1–2 days after surgery), two independent orthopedic researchers measured the shortest vertical distance (x) between the outermost limit of the ventral cortical bone resection and the medial wall of the intervertebral foramen (Figure 5). Interobserver reliability was analyzed using the intraclass correlation coefficient (ICC).

### 2.5. Statistical Analysis

Statistical analyses were performed using SPSS 25.0 (IBM Corp., Armonk, NY, USA). Continuous variables were tested for normality with the Shapiro–Wilk test. Normally distributed data were compared using paired *t*-tests, and non-normally distributed data using the Wilcoxon signed-rank test. Given the multiple comparisons of the four primary outcome measures (JOA, NDI, neck VAS, arm VAS) between preoperative and final follow-up only, Bonferroni correction was applied to control for Type I error. A corrected *p*-value < 0.0125 (0.05/4) was considered statistically significant. Interobserver reliability for CT measurements was assessed with a two-way random-effects ICC model (ICC > 0.75 indicates good reliability). Correlation analyses were performed to explore potential confounding factors; results are summarized in text and visualized in a heatmap (Figure 6).

## 3. Results

### 3.1. Demographic and Perioperative Data

A total of 40 eligible patients were ultimately included in this study. Table 1 summarizes the demographic characteristics of all patients. Except for disease duration, follow-up time, operative time, and postoperative hospital stay—presented as median (interquartile range) due to non-normal distribution—all other metrics are expressed as mean ± standard deviation. Among the 40 patients, 16 were male (40%) and 24 were female (60%). The mean age was 49.0 ± 10.6 years. The median disease duration was 4.5 (2.0, 36.0) months. The responsible levels were: C5-6 in 20 cases (50%), C6-7 in 19 cases (47.5%), and C7-T1 in 1 case (2.5%). The median follow-up time was 15.2 (12.2, 26.1) months.

### 3.2. Clinical Outcomes

All patients successfully underwent percutaneous endoscopic cervical keyhole surgery with anchored drill-assisted localization, with no cases requiring conversion to open surgery. The median operative time for the 40 patients was 70.0 (60.0, 80.0) minutes. Intraoperative blood loss was minimal (<10 mL) and difficult to quantify accurately in all cases. The median postoperative hospital stay was 3 (2, 3) days. Postoperative imaging confirmed resolution of nerve root compression in all patients (Figure 7). Correlation analysis was performed to assess potential confounding factors, including age, sex, BMI, disease duration, and comorbidities (e.g., diabetes mellitus, hypertension). Apart from expected correlations among symptom assessment indices, no significant associations were identified between preoperative demographic or clinical variables and postoperative improvement (Figure 6), suggesting that the observed clinical outcomes were not substantially influenced by the examined factors. However, due to the relatively small sample size, subgroup analysis of individual comorbidity effects was not feasible.

Table 2 summarizes the average shortest vertical distance (x) between the outermost boundary of the bone window’s operative area (the outermost aspect of the ventral cortical bone) and the medial wall of the intervertebral foramen, as measured postoperatively by two independent observers. Interobserver agreement for measuring distance “x” was excellent, with an intraclass correlation coefficient (ICC) of 0.92 (95% CI: 0.85–0.96). The distribution of x values was as follows: x ≤ −3.0 mm in 2 cases (5.0%); −3 < x < 0 in 5 cases (12.5%); 0.0 < x ≤ 3.0 in 22 cases (55.0%); and x = 0.0 in 11 cases (27.5%).

Paired-sample *t*-tests comparing preoperative VAS, JOA, and NDI scores revealed that all postoperative symptom assessment indices were significantly superior to preoperative values (Table 3), with statistically significant differences. According to the modified MacNab criteria, the excellent/good rate reached 97.5% (39/40), comprising 11 cases rated as excellent (27.5%), 28 as good (70%), and 1 as poor (2.5%) (Figure 8).

Correlation analysis was performed to assess potential confounding factors. Apart from expected correlations among symptom assessment indices, no significant associations were identified between preoperative demographic or clinical variables and postoperative improvement, suggesting that the observed clinical outcomes were not substantially influenced by the factors examined (Figure 6).

### 3.3. Complications

No intraoperative major complications—such as dural tear, nerve root injury, or vertebral artery injury—occurred. No cerebrospinal fluid leakage was observed. No symptomatic epidural hematoma requiring evacuation or surgical site infection was observed.

Minor complications were recorded as follows: transient postoperative axial neck pain occurred in 3 patients (7.5%), which resolved with conservative management within 2–4 weeks; persistent axial neck pain occurred in 1 patient (2.5%), which was managed with symptomatic treatment.

One patient (2.5%) reported a poor outcome, with worsened VAS and NDI scores at follow-up. This patient exhibited higher preoperative pain and disability scores compared to the cohort average.

## 4. Discussion

In this technical report, we describe a novel anchored drill-assisted localization technique for percutaneous endoscopic cervical keyhole surgery and present preliminary outcomes from 40 patients with single-level CSR. The findings demonstrate that patients treated with this technique exhibited significant postoperative improvements in VAS, JOA, and NDI scores (Figure 9), with an overall excellent/good rate of 97.5%. These results are comparable to those reported in previous studies [8,9,10,11,12,15,16], suggesting that this technique is feasible and achieves clinical outcomes consistent with established methods. Although no severe complications such as vertebral artery or nerve root injury occurred in this series, their potential risks require objective assessment. The minor complication rate (transient axial neck pain in 7.5%, persistent axial neck pain in 2.5%) was within the range reported in the literature for posterior cervical endoscopic procedures [11,12].

One patient in our cohort had a suboptimal outcome. A review of this patient’s data revealed that both preoperative pain and functional disability scores (VAS 6, NDI 62%) were higher than the group average, suggesting a potentially more complex pain phenotype. Initial symptom relief was followed by recurrence, manifesting as activity-related exacerbation of cervicobrachial pain. Therefore, despite imaging-confirmed adequate decompression, symptom recurrence could be attributed to potential factors such as perineural fibrosis, postoperative scar tissue stimulation, or psychosocial influences. We further postulate that symptom fluctuation may also be related to the extent of intraoperative disc decompression and the integrity of the annulus fibrosus, factors that could influence segmental stability.

Intraoperative localization poses significant challenges in endoscopic posterior cervical keyhole surgery. The limited endoscopic field of view and spatial distortion due to projection angles make structure identification particularly difficult. Consequently, most surgeons choose the interlaminar “V” point as the initial landmark [16,17,18,19,20,21]. While this method aids in rapid and accurate targeting of the segment, confirming this initial point and instrument position often necessitates frequent reliance on intraoperative fluoroscopy. Furthermore, if the surgeon is insufficiently familiar with anatomical depth or encounters anatomical variations, the initial localization starting from the “V” point may drift to adjacent segments due to changes in patient positioning or operative angles, again requiring repeated fluoroscopy and significantly increasing the patient’s radiation exposure risk [22,23,24,25]. The anchored drill-assisted cervical keyhole technique employed in this study addresses this by creating an artificial bony marker at the superolateral edge of the pedicle of the caudal vertebra in the target segment. Probing medially and superiorly from this point allows for rapid and accurate identification of the target interlaminar “V” point. In our series, the median number of fluoroscopic shots was 5 during the localization process, with no additional fluoroscopy required thereafter.

Regarding the preset operative trajectory, with the anchor point serving as the inferolateral boundary, all decompression maneuvers are strictly confined to its medial and cephalad aspects. This naturally creates an obliquely superomedial safe operative space in three dimensions, establishing a “safety boundary” for decompression. This boundary may help prevent the surgeon from blindly enlarging the bone window to expand the decompression range due to a lack of clear operative limits, thereby potentially avoiding excessive facet joint resection and the potential for subsequent segmental instability [5,26,27]. Additionally, vertebral artery injury is a potentially catastrophic complication of posterior cervical endoscopic surgery. Studies indicate an incidence ranging from 4.1% to 8.2% in posterior approaches [13,14], which is a significant deterrent for many surgeons. From an anatomical perspective, the anchor point and the medial border of the vertebral artery lie on the same vertical line (Figure 10). Postoperative CT measurements (Table 2) showed that only 55% of cases maintained a measurable safe distance (x > 0) between the bone window projection and the medial wall of the intervertebral foramen. Crucially, no vertebral artery injuries occurred in the remaining 45% of cases (x ≤ 0, including 17.5% with x < 0).

We acknowledge that the two-dimensional CT measurement (x) alone does not fully capture three-dimensional surgical safety. The absence of vertebral artery injuries in our series is attributable to the inherent three-dimensional trajectory of the procedure: even when the two-dimensional projection appears to “touch” or “cross” the medial foramen wall, the actual surgical instruments—following the preset oblique superomedial trajectory from the anchor point—remain safely within the neural foramen and maintain a safe distance from the vertebral artery located in the anterior transverse foramen. This conceptual distinction between two-dimensional projection and three-dimensional instrument positioning is central to the safety rationale of this technique.

The anchored drill features a non-threaded tip with a depth-limiting structure. Although non-threaded temporary pins have been reported to migrate in other spinal procedures, we observed no cases of drill displacement in this series. Several factors likely contributed to this favorable outcome: cortical bone engagement of the tip, gentle tapping technique, and—most importantly—the brief intraosseous retention time (typically 1–2 min), as the drill was removed immediately after establishment of the working channel. The working cannula then independently maintained channel stability throughout the remainder of the procedure.

The feasibility of this technique across different cervical levels warrants comment. Although the majority of cases in this series involved C5–C6 and C6-C7, the anatomical principle—placement of the anchored drill at the superolateral edge of the caudal vertebral pedicle—is applicable to levels from C3-C4 through C7-T1, provided the pedicle anatomy is clearly identifiable on intraoperative fluoroscopy.

### Limitations and Future Directions

As a preliminary technical report without a control group, our study cannot definitively establish the effectiveness of the technique. However, the consistent significant improvements in all clinical outcome measures (VAS, JOA, NDI) from preoperative to final follow-up, combined with imaging-confirmed decompression in all patients, provide internal evidence supporting the preliminary effectiveness of the anchored drill-assisted technique.

This study has several limitations. First, this is a single-center retrospective case series without a control group. The inherent selection bias and information bias of retrospective studies preclude direct causal inference between the technique and clinical outcomes. As a technical report, our findings should be considered preliminary and hypothesis-generating rather than definitive. Second, the sample size is relatively small (n = 40) and was determined by consecutive cases rather than formal power calculation, which is acceptable for an exploratory preliminary report but still restricts generalizability and precludes subgroup analyses, and the median follow-up of 15.2 months (range, 12.2–26.1 months) is insufficient to assess long-term outcomes such as segmental stability, progressive facet degeneration, or delayed biomechanical consequences. Third, all procedures were performed by a single senior spine surgeon at a single institution. Given the single-operator design, inter-operator variability was not assessed. This limits the assessment of inter-operator reproducibility and generalizability of the technique to other surgical settings. Future multicenter studies involving multiple surgeons are needed to validate the reproducibility of this technique. Fourth, the technique requires the surgeon to be familiar with the anatomical landmarks and the specific anchored drill device, which may limit generalizability. Fifth, while we reported the number of fluoroscopic shots, we did not quantify the actual radiation dose (in mSv) received by patients and surgical staff. Future studies should include formal radiation dosimetry to better assess the radiation safety profile of this technique.

Future research should focus on two main areas: first, conducting prospective, multicenter, large-sample randomized controlled trials to provide further evidence-based medical support; second, performing postoperative biomechanical analyses of the cervical spine to clarify the impact of this technique on cervical stress distribution and stability, thereby verifying its long-term advantages.

## 5. Conclusions

The anchored drill-assisted localization technique represents a meaningful refinement of posterior percutaneous endoscopic cervical keyhole surgery. In this preliminary technical report, the technique demonstrates favorable safety and efficacy in treating single-level cervical spondylotic radiculopathy. By providing a stable intraosseous reference, it may enhance spatial orientation, potentially reduce reliance on intraoperative fluoroscopy, and establish a clear, reliable safety boundary for decompression. Prospective comparative studies are needed to validate these preliminary findings and to determine whether this technique offers advantages over conventional approaches, as the current preliminary report lacked a control group for direct comparison.

## Figures and Tables

**Figure 1:**
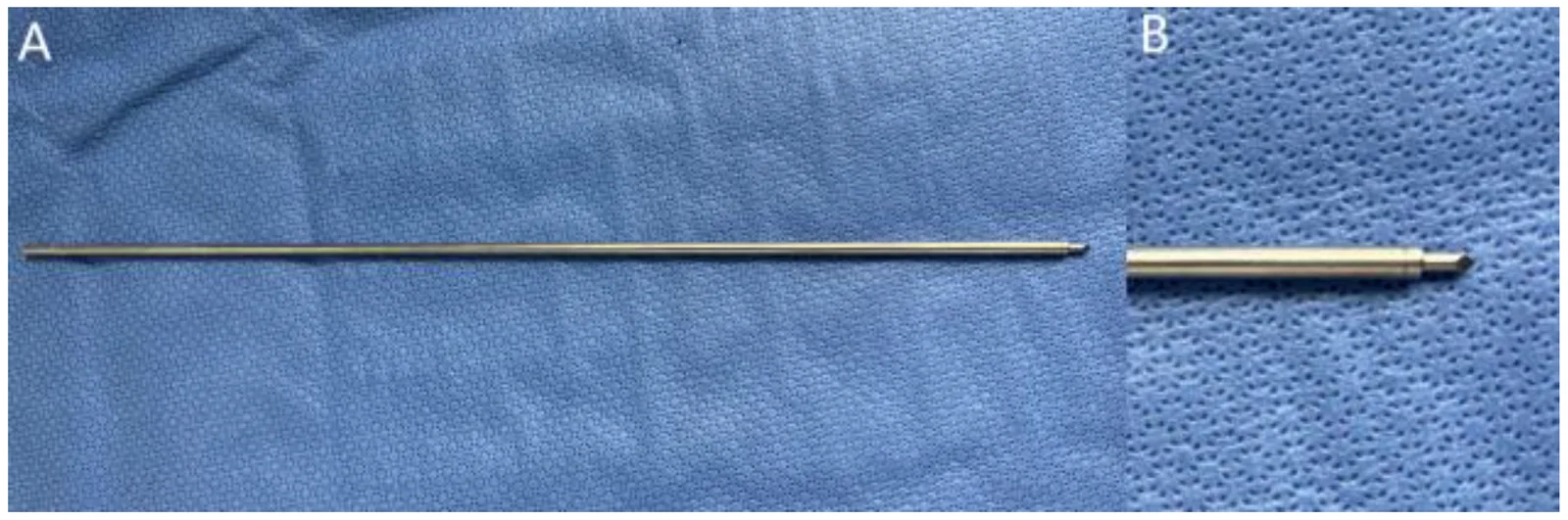
Design and dimensions of the custom-designed anchored drill. (**A**) Overall view of the anchored drill. (**B**) Close-up view of the drill tip, which is 4 mm high and 2 mm in diameter, featuring a depth-limiting safety structure.

**Figure 2:**
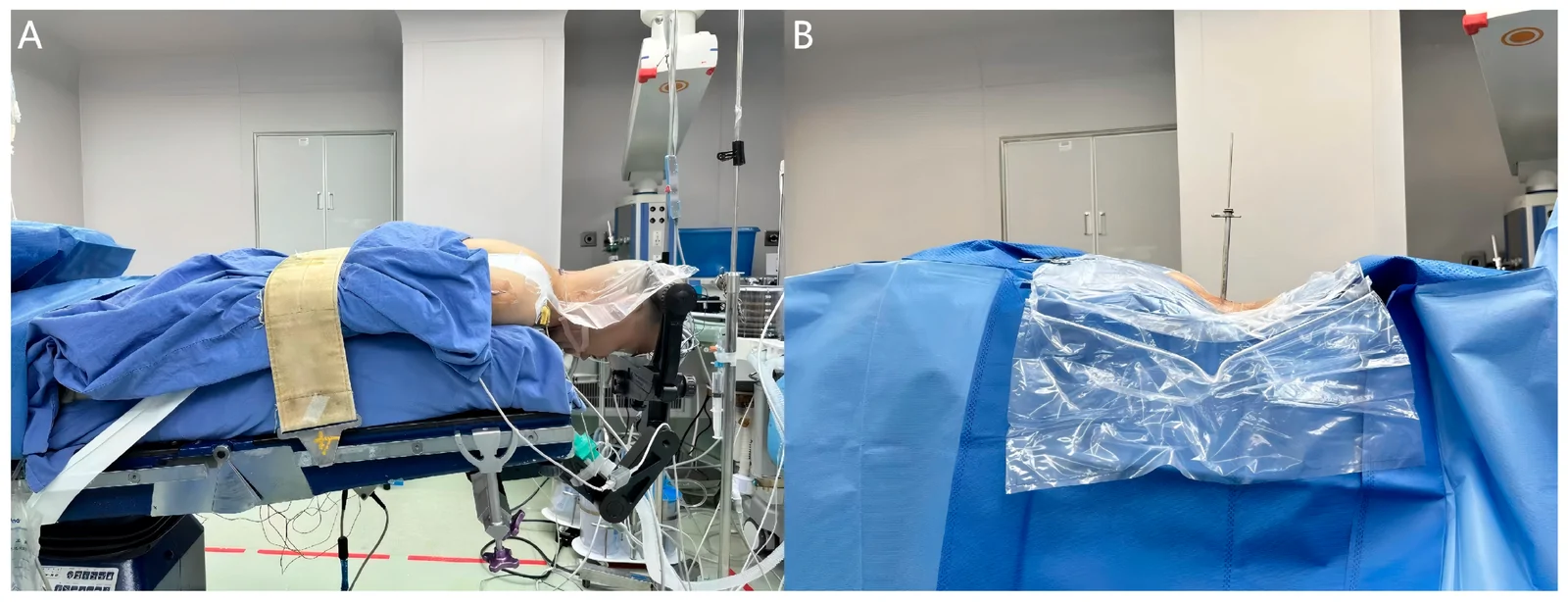
(**A**) Patient positioning: The lamina of the index level should be parallel to the floor; (**B**) Maintain the long axis of the anchored drill perpendicular to the plane of the lamina.

**Figure 3:**
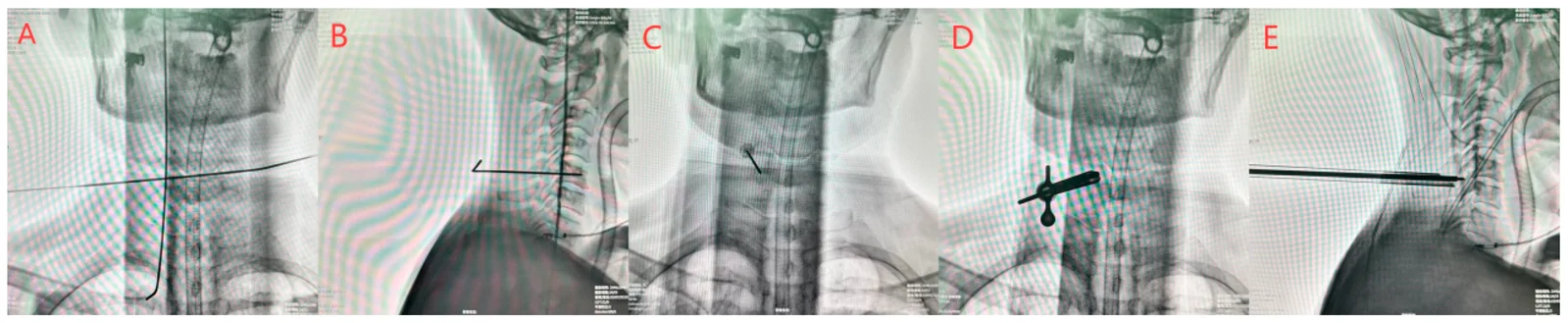
Fluoroscopically guided steps for anchored drill implantation. (**A**,**B**) Anteroposterior and lateral views confirming the target level. (**C**) Puncture needle tip reaching the junction of the lamina and lateral mass of the caudal vertebra. (**D**,**E**) Final anteroposterior and lateral views verifying accurate placement of the anchored drill at the superolateral edge of the C6 pedicle.

**Figure 4:**
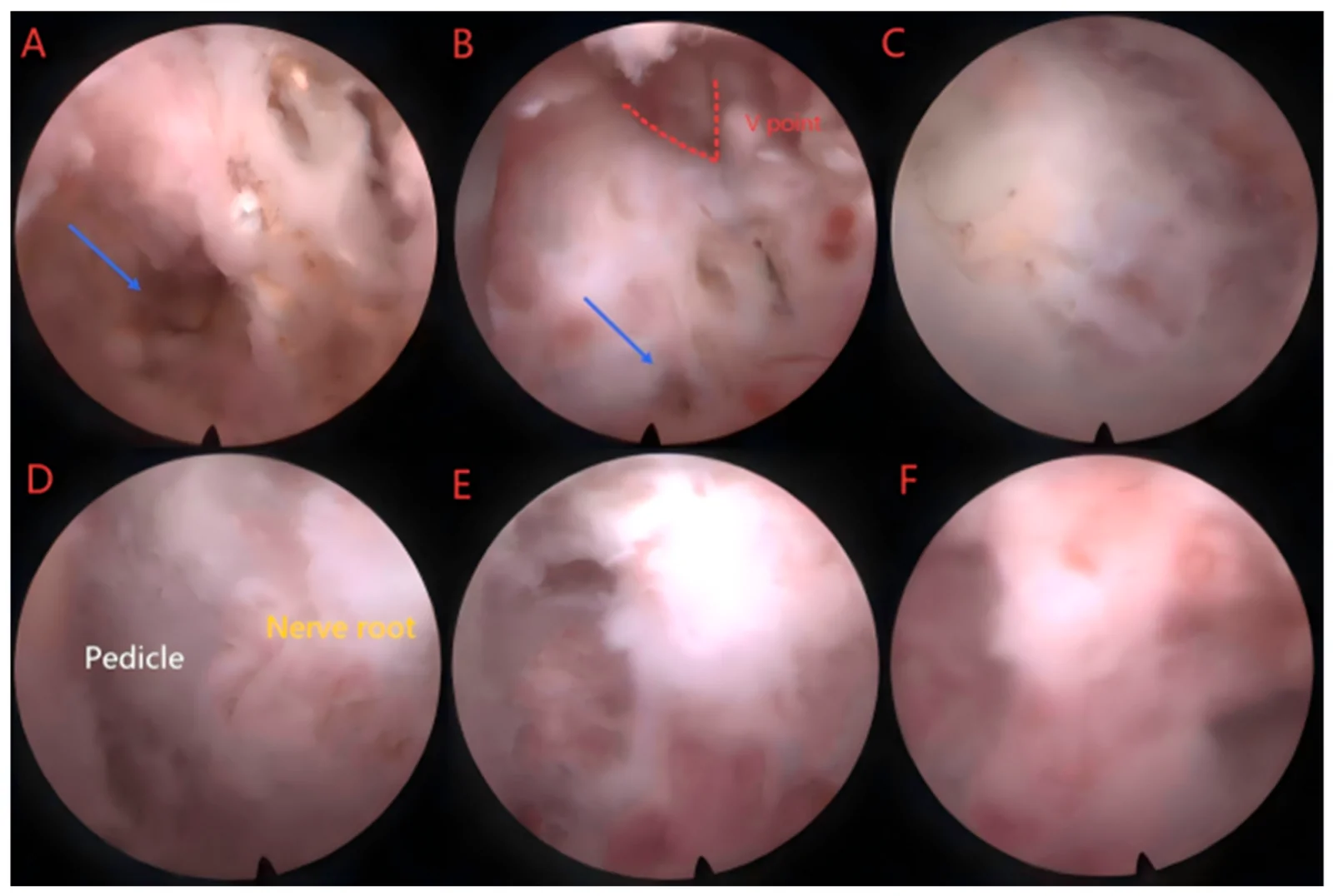
(**A**,**B**) Blue arrow indicates the “anchor point”; using the anchor point as a reference to locate the “V” point of the target segment; (**C**) C5 laminotomy performed up to the insertion of the ligamentum flavum; (**D**) C6 laminotomy performed until exposure of the pedicle; (**E**) Decompression of the nerve root axilla along the medial border of the pedicle; (**F**) Overall decompression effect after shoulder decompression of the nerve root.

**Figure 5:**
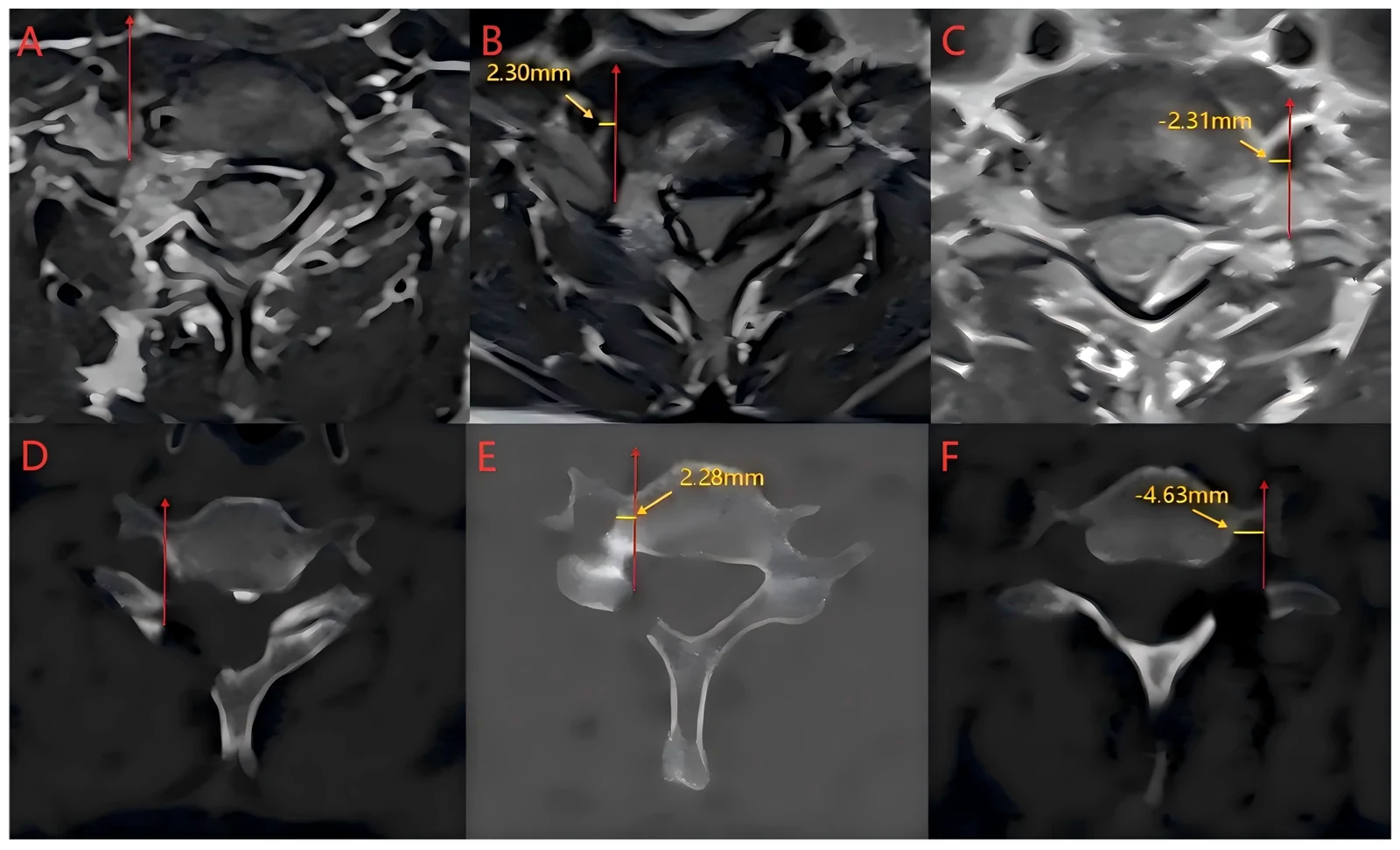
Imaging measurement method for the safety boundary. (**A**–**C**) Schematic diagram and representative T2-weighted MRI axial images showing the vertical distance (x = yellow line) between the outermost boundary of the decompression corridor (red arrow) and the medial wall of the intervertebral foramen. (**D**–**F**) Corresponding postoperative CT axial images demonstrating actual measurements in three representative scenarios: x > 0 (safe distance), x = 0 (at the boundary), x < 0 (beyond the boundary, yet clinically safe due to the oblique trajectory).

**Figure 6:**
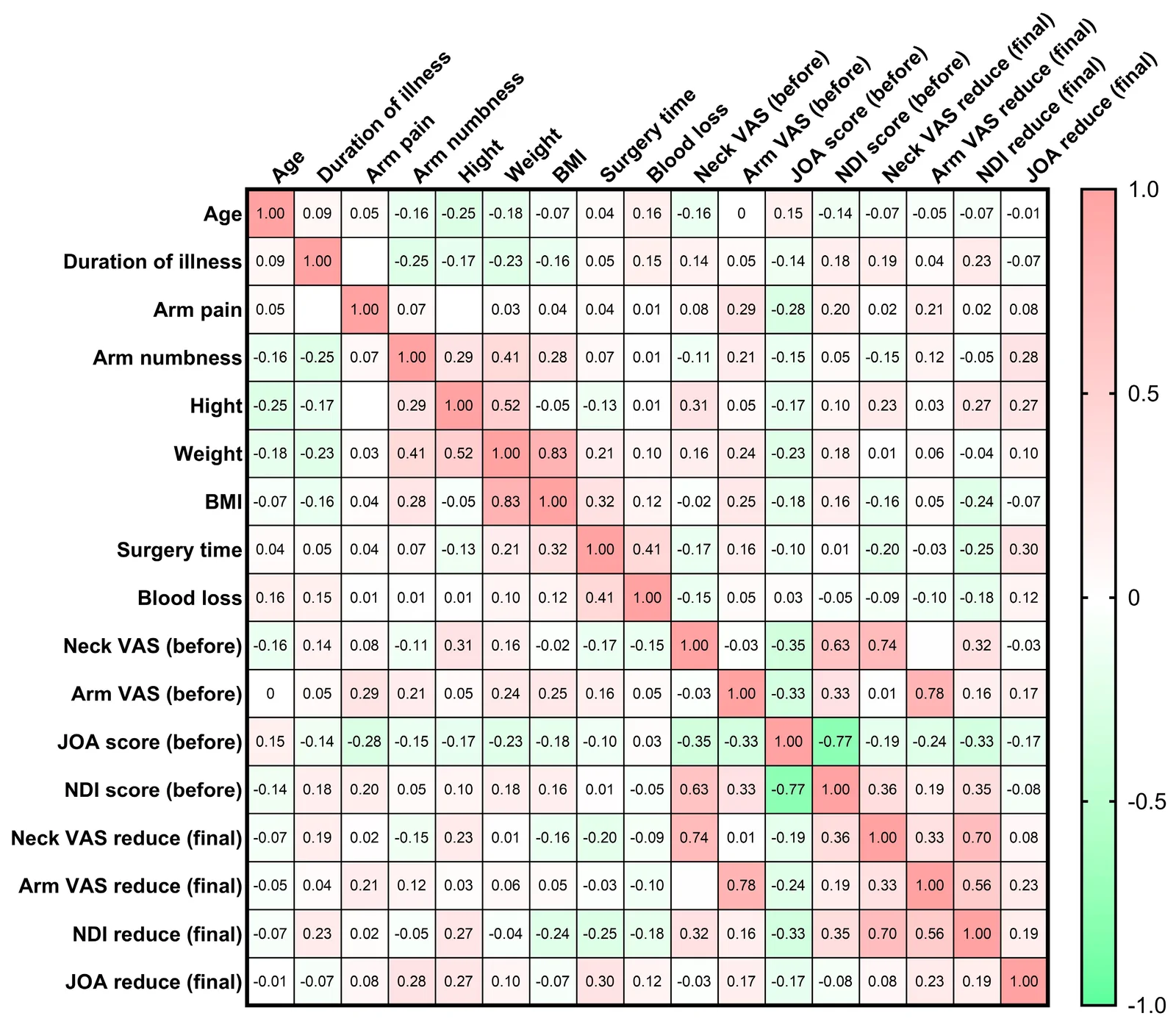
Correlation heatmap between patient-related indicators and postoperative outcomes. Apart from expected correlations among symptom assessment indices, no significant associations were identified.

**Figure 7:**
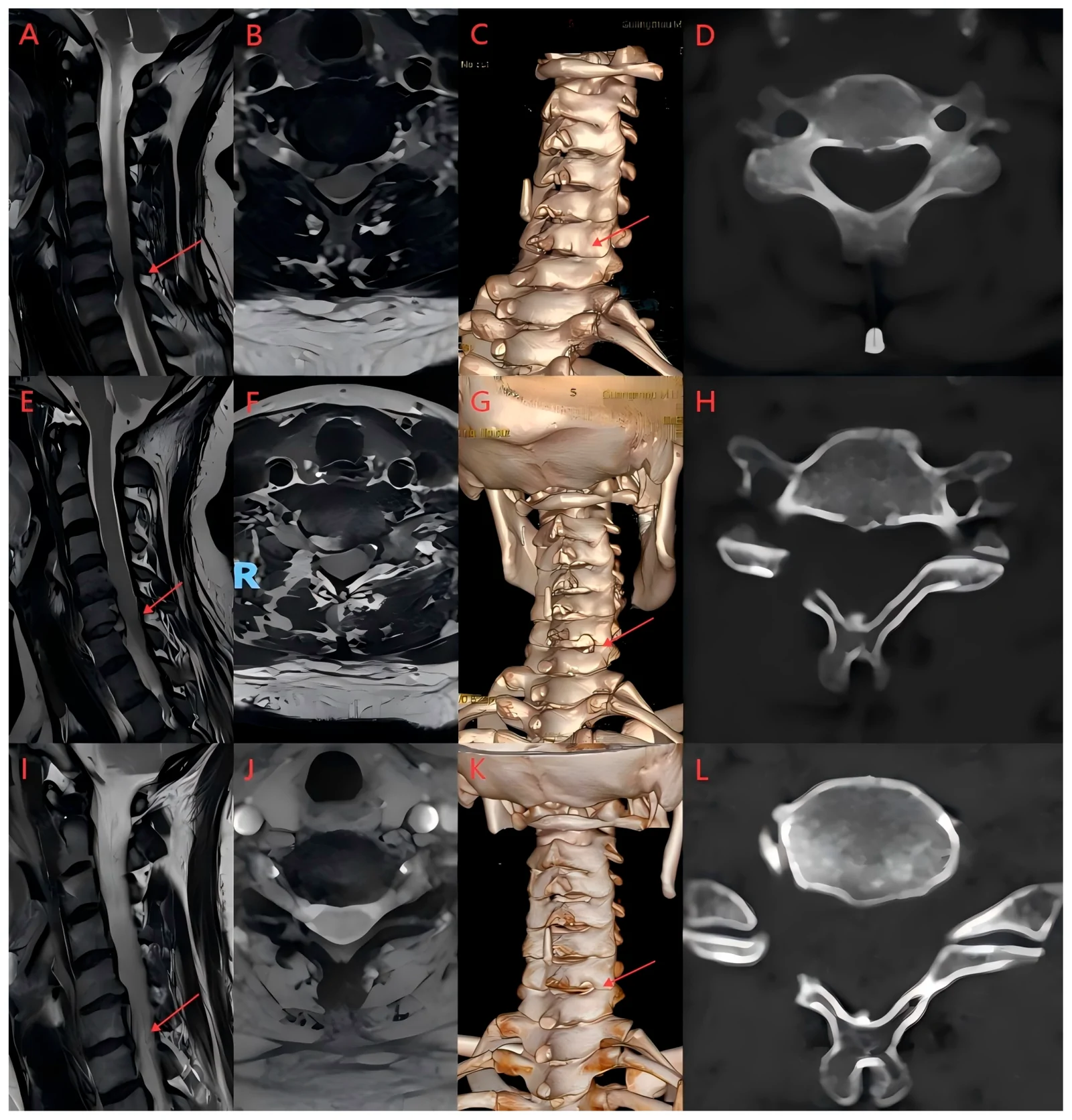
(**A**,**B**) Preoperative MRI showing cervical disc herniation; (**C**,**D**) Preoperative CT 3D reconstruction showing intact bony structures; (**E**,**F**) Postoperative MRI showing thorough ventral decompression of the nerve root; R, right side. (**G**,**H**) Postoperative CT 3D reconstruction showing most of the facet joint preserved after keyhole decompression; (**I**,**J**) 2-year postoperative MRI showing good status of the surgical segment with fading surgical traces; (**K**,**L**) 2-year postoperative CT 3D reconstruction showing a healing trend of the keyhole bone window with significant reduction in the size of the bony defect. Red arrows indicate the site of nerve root compression. The same site is indicated in the postoperative and follow-up images to demonstrate the decompression effect.

**Figure 8:**
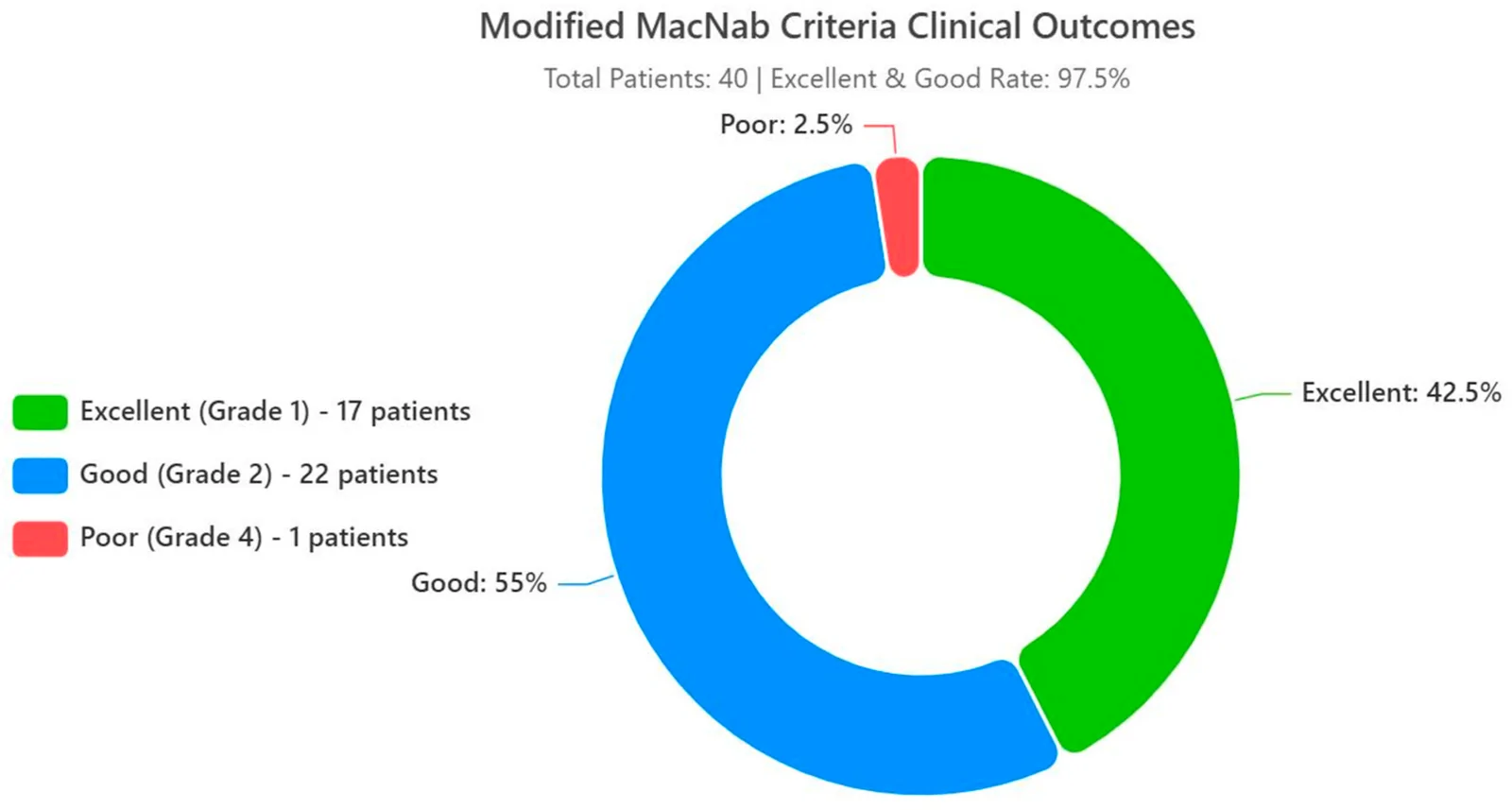
Distribution of modified MacNab outcomes. The excellent/good rate was 97.5% (39/40), comprising 11 excellent (27.5%), 28 good (70%), and 1 poor (2.5%).

**Figure 9:**
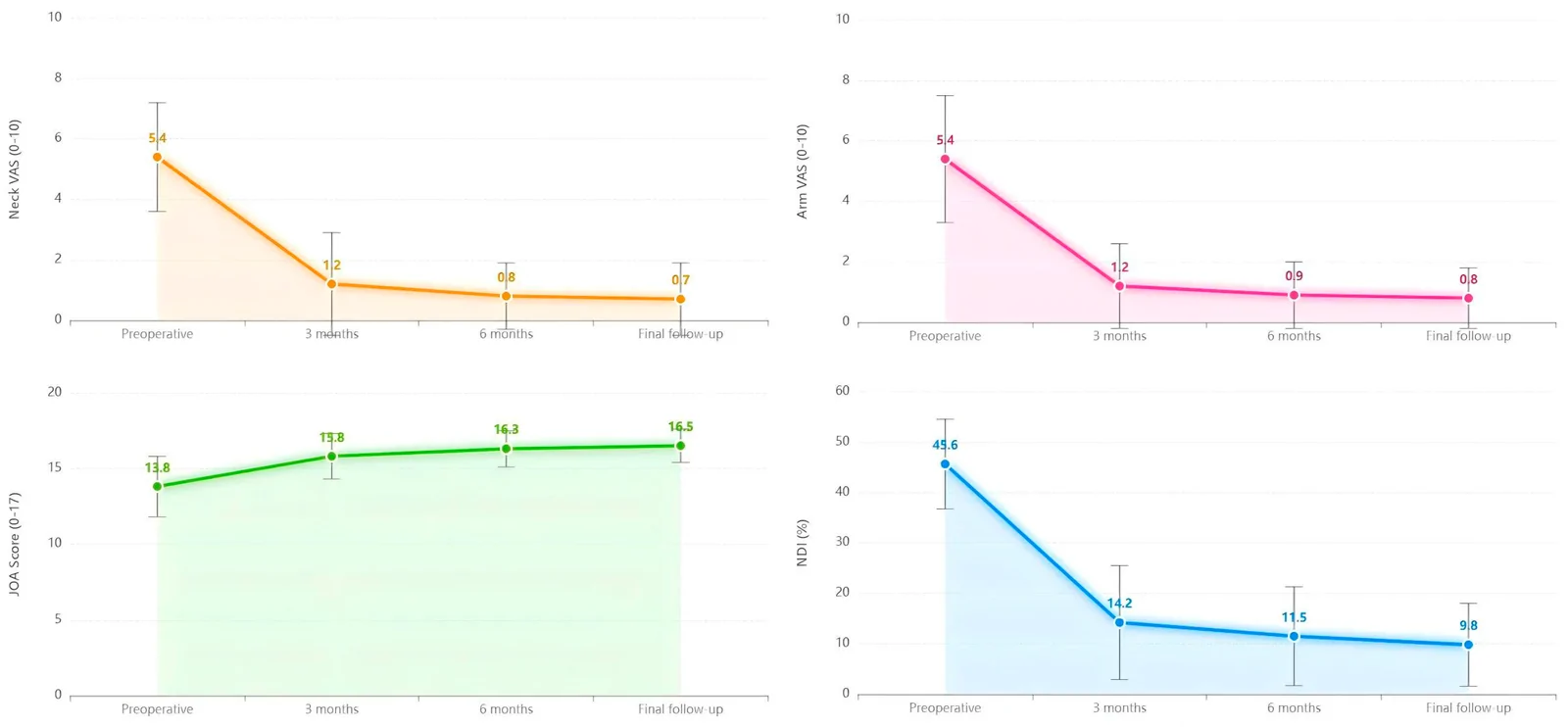
VAS, JOA, and NDI scores showed significant improvement compared to preoperative values. Error bars represent standard deviation.

**Figure 10:**
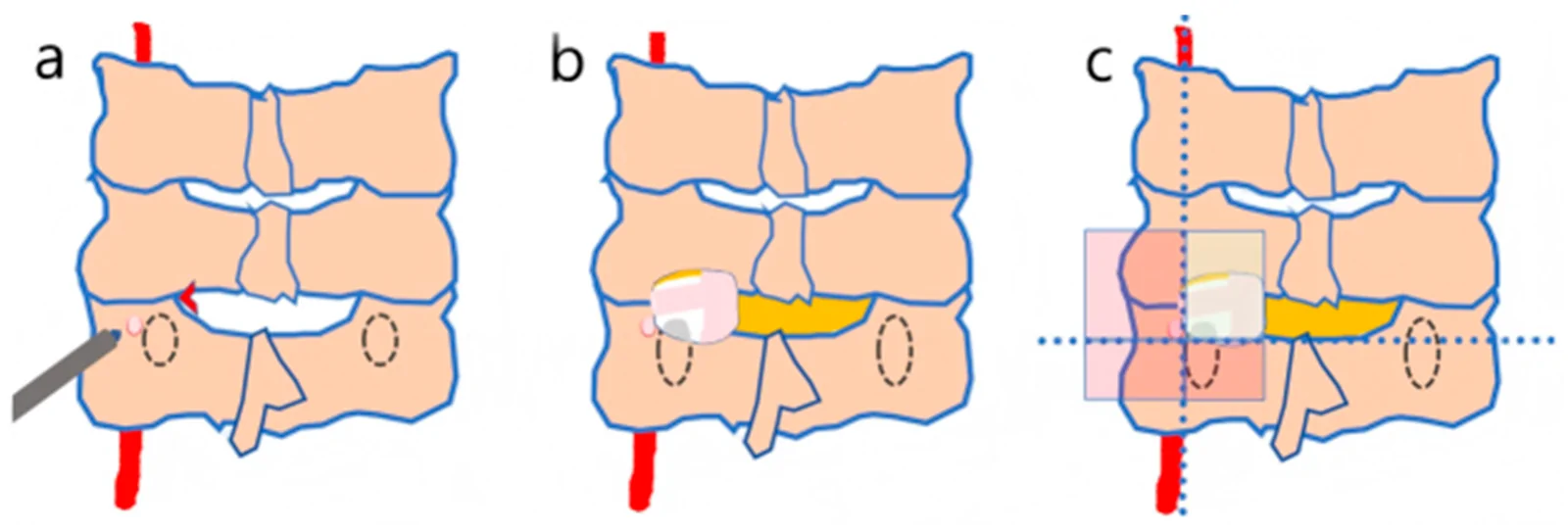
Conceptual diagram illustrating the safety boundary principle. (**a**) The anchored drill is placed at a consistent anatomical landmark: the superolateral edge of the pedicle of the caudal vertebra. (**b**) All bone removal (shaded area) is performed medial and cephalad to this reference point. (**c**) This constructs a three-dimensional safe operative zone (blue dashed lines), whose projection maintains a safe distance from the vertebral artery (red) located within the transverse foramen. The black dashed circle indicates the pedicle.

**Table 1:** Demographic, clinical, and surgical characteristics of patients (n = 40).

Variable	Value
**Demographic data**	
Age (years)	49.0 ± 10.6
Sex, n (%)	
Male	16 (40.0)
Female	24 (60.0)
BMI (kg/m^2^)	24.3 ± 3.1
Disease duration (months) †	4.5 (2.0, 36.0)
Follow-up duration (months) †	15.2 (12.2, 26.1)
**Surgical level, n (%)**	
C5-6	20 (50.0)
C6-7	19 (47.5)
C7-T1	1 (2.5)
**Intraoperative and postoperative data**	
Operative time (min) †	70.0 (60.0, 80.0)
Estimated blood loss (mL) †	10.0 (5.0, 20.0)
Postoperative hospital stay (days) †	3.0 (2.0, 3.0)
Cerebrospinal fluid leakage, n (%)	0 (0.0)
Vertebral artery injury, n (%)	0 (0.0)
Transient Axial Neck Pain	3 (7.5)
Persistent Axial Neck Pain	1 (2.5)

Data are presented as mean ± SD for normally distributed continuous variables, median (interquartile range) for non-normally distributed continuous variables, and n (%) for categorical variables. † Non-normally distributed variable (Shapiro–Wilk test, *p* < 0.10). BMI, body mass index.

**Table 2:** Distance from Bony Window to Foramen.

Safety Distance Group (mm)	CT Case Number (n = 40)	CT Percentage (%)
x ≤ −3.0	2	5.0%
−3 < x < 0	5	12.5%
x = 0.0	11	27.5%
0.0 < x ≤ 3.0	22	55.0%

**Table 3:** Clinical outcomes at preoperative and postoperative time points (n = 40).

Time Point	JOA Score	NDI (%)	Neck VAS	Arm VAS
Preoperative	13.8 ± 2.0	45.6 ± 8.9	5.4 ± 1.8	5.4 ± 2.1
3 months postoperatively	15.8 ± 1.5	14.2 ± 11.3	1.2 ± 1.7	1.2 ± 1.4
6 months postoperatively	16.3 ± 1.2	11.5 ± 9.8	0.8 ± 1.1	0.9 ± 1.1
Final follow-up	16.5 ± 1.1	9.8 ± 8.2	0.7 ± 1.2	0.8 ± 1.0
*p*-value (preop vs. final) ‡	<0.001	<0.001	<0.001	<0.001

Data are presented as mean ± SD. JOA, Japanese Orthopaedic Association; NDI, Neck Disability Index; VAS, Visual Analog Scale. ‡ Paired *t*-test for JOA and NDI; Wilcoxon signed-rank test for neck and arm VAS. *p*-values were adjusted using Bonferroni correction for the four comparisons between preoperative and final follow-up (corrected significance threshold: *p* < 0.0125). All comparisons remained statistically significant after correction (all adjusted *p* < 0.001).

## Data Availability

The datasets generated and analyzed during the current study are not publicly available due to patient privacy concerns but are available from the corresponding author on reasonable request.

## list of Abbreviations

ACDF	Anterior Cervical Discectomy and Fusion
ASD	Adjacent Segment Degeneration
BMI	Body Mass Index
CSR	Cervical Spondylotic Radiculopathy
CT	Computed Tomography
ICC	Intraclass Correlation Coefficient
JOA	Japanese Orthopaedic Association
MRI	Magnetic Resonance Imaging
NDI	Neck Disability Index
PCF	Posterior Cervical Foraminotomy
VAS	Visual Analog Scale
